# Hydrogel Applicability for the Industrial Effluent Treatment: A Systematic Review and Bibliometric Analysis

**DOI:** 10.3390/polym15112417

**Published:** 2023-05-23

**Authors:** Luis Enrique Flores-Valenzuela, José Vulfrano González-Fernández, María Verónica Carranza-Oropeza

**Affiliations:** Facultad de Química e Ingeniería Química, Universidad Nacional Mayor de San Marcos, Lima 15081, Peru

**Keywords:** hydrogels, adsorption, industrial effluent treatment, dyes removal, heavy metal ion adsorption, fixed-bed columns, systematic review

## Abstract

In recent decades, hydrogels, as adsorption materials, have received important attention due to their characteristics and properties, such as mechanical strength, biocompatibility, biodegradability, swellability, and stimuli sensitivity. In the actual framework of sustainable development, it has been imperative to develop practical studies of hydrogels in the treatment of actual industrial effluents. Accordingly, the current work has, as its objective, to make evident hydrogels’ applicability in the treatment of actual industrial effluents. For this purpose, a bibliometric analysis and systematic review based on the PRISMA (Preferred Reporting Items for Systematic Reviews and Meta-Analyses) method were conducted. The relevant articles were selected from the Scopus and Web of Science databases. Some important findings were that: (1) China is the leading country when it comes to hydrogel application in actual industrial effluents, (2) the motor studies are focalized on the treatment of wastewater by hydrogels, (3) the fixed-bed columns are suitable unit equipment for the treatment of industrial effluents of using hydrogels, and (4) the hydrogels show excellent adsorption capacities of ion and dye contaminants present in industrial effluents. In summary, since the implementation of sustainable development in 2015, the progress of practical hydrogel applications in the treatment of industrial effluent has been receiving more attention, and the selected studies demonstrate the implementation viability of these materials.

## 1. Introduction

The dumping of industrial wastewater, without previous treatment, into water bodies is an important source of environmental contamination. In particular, contamination through chemical products presents a public, environmental, and global preoccupation [1,2]. Discharging industrial effluents into rivers, lakes, rivulets, and wells could generate serious health problems for humans, animals, and the ecosystem [3].

Water consumption always means contamination of itself since when someone or some process (chemical, cosmetic, plastic, cement, agrochemical, textile, etc.) uses water, it will always be contaminated in the end; those industries generate big volumes of wastewater with different types of contaminants, which must be treated before dumping them in the environment [4,5,6,7]. Based on this premise, countries are aware of it and are taking political steps to protect hydric resources [8,9].

Over the years, countries have taken normative and legislative actions inside their territory, establishing and demanding the treatment of wastewater coming from any industrial sector before its final disposal in water bodies. For instance, Europe since the 1990s has developed committees for the generation of directives to implement zero wastewater discharge [4,10]. An important development on a global level happened on 25 September 2015 at a conference of the United Nations for sustainable development, where an agenda was established regarding production changes and waste management [11,12]. Among the sustainable development goals (SDG), seven goals have the management and valorization of water and waste as their main topic.

Over the years, traditional methods for treating industrial wastewater have been converted into unreliable, obsolete, and inefficient technologies due to the novel characteristics and properties that current contaminants possess [13]. Thus, there is a need to generate new technologies and materials whose adsorption capacity for contaminants is easy to operate, economical, nontoxic, and effective.

Over the years, many methods have been applied to eliminate contaminants, such as chemical precipitation, adsorption, membrane filtration, ozonation, ionic exchange, microwave catalysis, biodegradation, photo-catalytic degradation, coagulation-flocculation, chemical oxidation, electrochemical degradation, and electro-flocculation [14,15,16,17,18,19,20,21,22,23]. However, most processes exhibit serious economic and technical difficulties in their application on an industrial scale [4,24] (Table 1).

Hydrogels are polymeric networks crosslinked and insoluble in water that are able to adsorb and keep a large amount of water in their structure. In addition, they are considered nontoxic materials, low cost, easy to prepare, eco-friendly and recyclable, and ideal for use as adsorbents of different elements or molecules. The tridimensional structure of hydrogels, whose polymeric chains and functional groups present a hydrophilic behavior able to adhere to some metallic ions or colorants for adsorption give them unique properties as adsorbent agents; hydrogels are principal candidates for contaminant removal from industrial effluents since their adsorption capacity could generate a large efficiency percentage; likewise, their control and reutilization give them advantages with respect to other adsorbents [25,26,27,28]. Most of the studies developed concerning hydrogels have focused on investigating the versatility of elements and compounds capable of being adsorbed by hydrogels, as well as improving their adsorption capacity under different environmental stimuli through the strengthening of their mechanical properties, incorporation of nanoparticles, and degree of biodegradability, among others. All of these studies have been conducted on a laboratory scale in a controlled environment with pre-established operating conditions. However, it is imperative to perform applicative studies to address the adsorption of the hydrogels in the treatment of current contaminants coming from industrial dumping since through these studies, the technical feasibility and environmental and economic impacts of hydrogels in the treatment of industrial pollutants can be evidenced. For these reasons, the present study carried out a systematic review to demonstrate the current state of the real applications of hydrogels in the industry and to show the audience the research and application scope that has been achieved in recent years.

Additionally, the present work has, as its target audience, professionals and researchers coming from the engineering area dedicated to the search for new technologies for the treatment of industrial effluents, which applies to the industrial level, to be viable in technical, economic, and environmental terms.

This article designed the research question based on the PICO strategy (P: problem; I: intervention; C: control; O: outcome) [29]. Consequently, the research question was: how effective are hydrogels as removal materials for the treatment of contaminants present in the actual industrial effluents? Furthermore, it established the goal: to evidence the applicability of hydrogels as adsorption materials to actual industrial effluents.

## 2. Materials and Methods

The present study was performed based on systematic review fundaments, and the relevant scientific evidence was evaluated and synthesized in accordance with PRISMA methodology (Preferred Reporting Items for Systematic Review and Meta-Analyses); likewise, the study realization lies in previous works with similar methodologies [30,31,32]. For the scientific article selections, principal databases about engineering were used: Scopus and Web of Science. In this way, the integrity and academic quality of the studies selected within a specific period of the years (2015–2022) were assured. The last consulted date was 26 October 2022. The literary research was conducted through a specific investigation using “titles”; likewise, the search codes were conformed to Boolean connectors for the prime source identifications. The keywords were compounded by the terms “Hydrogel” AND “Treatment OR Adsorption OR Water OR Tailing OR Effluent”, and the exclusion of some terms was made “Drug OR Cancer OR Tumor OR Delivery” (Table 2). The literary inquiry by “titles” allowed the recovery of the relevant articles, minimized sensibility loss, and gave thematic insight [33,34,35].

The retrieved articles from the literature search were analyzed using a bibliometric study (descriptive) to obtain metrics on publications, authors, countries, journals, and other parameters using the functions of the Bibliometrix R-package (http://www.bibliometrix.org (accessed on 13 November 2022) [36] of the free code software RStudio 4.2.1. [37] and through a systematic study based on the methodology PRISMA 2020 [38].

Subsequent to the bibliometric analysis, the articles’ evaluations were made as a function of their pertinence to answer the investigative question. The Rayyan virtual platform was employed [39] as a first review of the articles regarding titles and abstracts, selecting articles whose topics were in concordance with the investigative question. Subsequently, an exhaustive review was made based on eligibility criteria, individual contribution studies, and aggregated results, which contribute new information about the main theme. All of this was done with the clear goal of minimizing bias.

The eligibility criteria (inclusion and exclusion) for the selection of relevant articles were made impartially and independently.

- Inclusion criteria
  - Studies in the final phase of publication;
  - Scientific articles;
  - Study period between 2015–2022;
  - Works focused on the treatment of industrial wastewater through hydrogels as adsorption technologies;
  - Publications in the English language.
- Exclusion criteria
  - Titles that do not have at least two keywords;
  - Other languages;
  - Review articles;
  - Thesis, dissertations, books, conferences;
  - Grey literature.

**Figure 1:**
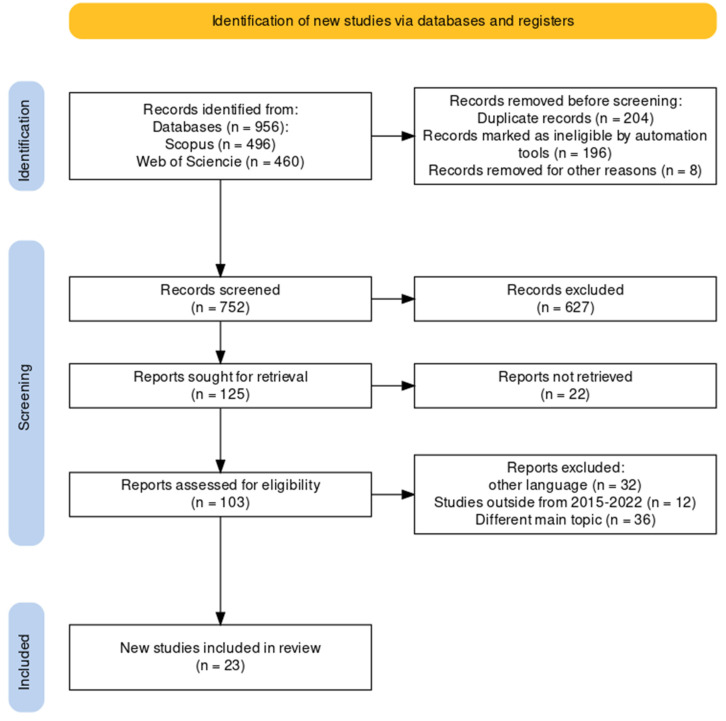
Flow diagram (PRISMA) of identified articles. Own elaboration based on reference [38].

The flow diagram for the included article selection in the systematic review based on the PRISMA methodology [40,41,42] is shown in Figure 1.

## 3. Results

The established study period (2015–2022) was chosen based on a significant global event, the United Nations Sustainable Development Summit, held in New York. This summit established guidelines for sustainable development regarding the three pillars, the environment, economy, and society.

These guidelines have environmental and social motivations because of global issues, such as global warming, famine, water scarcity, and discrimination. In this congress, 17 sustainable development goals were established for poverty eradication, hydric and biology sources protection, and human prosperity assurance without prejudice for the future generations’ sources (Agenda 2030) [43].

Based on this, a bibliometric study was carried out to determine the direction of last year’s studies and present the principal countries, authors, and journals regarding the development of hydrogels as adsorption materials. Likewise, through the bibliometric analysis, the impact of this milestone in the scientific community and how it has boosted the production of scientific articles were visualized; specifically for this study, articles related to the implementation of new technologies, such as hydrogels, for industrial wastewater treatment, were used.

### 3.1. Investigative Field Evolution

#### Literature Development from 2000–2022

**Figure 2:**
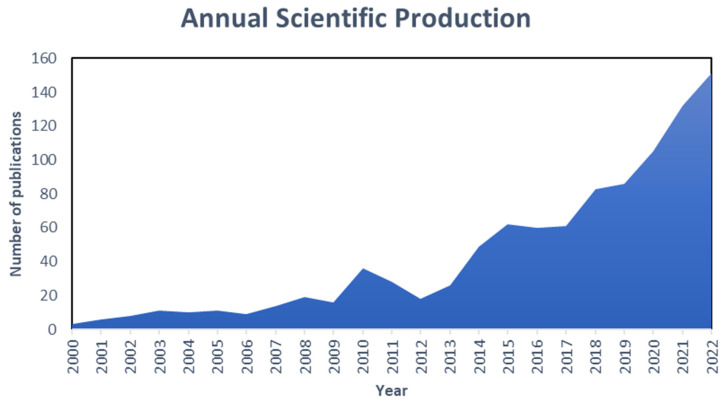
Annual scientific publications from 2000 to 2022 on applicative hydrogels in the treatment of industrial effluents. Note: Information was collected from Scopus and Web of Science databases.

A relevant indicator of scientific advance regarding a specific theme is the number of published articles. Figure 2 shows the number of annual publications about the thematic “hydrogels applicative studies on the treatment of industrial wastewater” in the period from 2000 to 2022. In addition, this graphic arrangement allowed us to obtain quantitative information concerning scientific production and the annual increase rate.

The studies concerning hydrogels and their applications as adsorption materials in the treatment of industrial effluents had few relevant degrees and production for the first 10 years of the 21st century. However, at the beginning of 2012, the United Nations congregated a conference about sustainable development (Rio+20) [44] whose goal was concretizing a renewed political commitment for sustainable development, assessing the achieved progress and addressing new challenges, so that the 192 member countries signed a commitment to boost the established goals inside their territorial framework; as a consequence, the scientific production regard to hydrogels grew significantly, and this fact was possible due to the characteristics of hydrogels that make them environmentally viable, economically attractive, and technologically efficient in technologies in concordance with the agreement established in Rio+20. The next years until 2015 maintained an important production of articles inside the scientific community for their properties and applications in different areas, such as cosmetics [45], personal care products [46], food [47,48], plastics [49,50], agriculture [51,52], sensors [53,54], etc. Afterwards, in 2016–2017 scientific article production fell slightly, and at the same time, the established agreements in the United Nations Sustainable Development Summit entered into force; however, this descent can be considered an impulse drive because in the subsequent years 2018–2022, studies on hydrogels as adsorption materials for the treatment of industrial effluents were in ascent, gaining a higher production than previous years.

Specifically, in 2019, we did not appreciate a significant advance in hydrogels and their industrial applications; nevertheless, in the subsequent years (2020–2021), scientific production grew considerably, above all, in 2021. These affirmations are interesting due to the global situation, the COVID-19 Pandemic, in which schools, universities, development, and investigation centers were closed. At present (2022), 151 scientific articles have been registered, which is higher than previous years (2021: 132 articles, 2020: 105 articles, 2019: 86 articles).

On the other hand, the most relevant keywords for each study year were selected, through which a first image about the annual predominated theme in the period 2015–2022 was generated. Then, it was observed in the last seven years that the articles have two keywords that are the most recurrent: the first makes reference to the study object (hydrogels), and the second makes reference to the application of the study subject (adsorption). With these two terms, it was possible to make a first global concept about the thematic of hydrogels address in the selected articles.

In addition, particular keywords for each study year showed which specific study approach was developed in that year. Thus, in the 2015–2017 period, we had the following recurrent keywords: adsorption (41), hydrogel (41), kinetics (11), chitosan (7), heavy metal ions (6), swelling (6), adsorption kinetics (5), adsorption mechanism (5), and cellulose (5). The most relevant authors were as follows: Wang Y., Wang X., Zhang Y., Liu Y., Wang J, Li J., Wang H., Li Y., and Li X. The theme addressed by those articles in this period of years was focused on natural-based hydrogel synthesis and their ion heavy metal adsorption capacities. After this period of years, in 2018–2019, the published works were principally compounded by the following keywords: hydrogel (48), adsorption (43), chitosan (8), alginate (6), heavy metal ions (6), and methylene blue (5). The predominant authors in this lapse of time were Zhang Y., Cui W., Liu L., Liu Y., Zhu Y., Liang Y., Ma J., Wang X., An W., and Liu C. Based on the keywords, this period addressed themes similar to the period 2015–2017, except for the keyword methylene blue, which referred to the studies about industrial dyes. The remaining four years (2019–2022) of scientific production were represented by the following keywords: hydrogel (98), adsorption (85), chitosan (23), sodium alginate (17), graphene oxide (14), kinetics (9), desalination (7), polyacrylamide (7), water treatment (7), adsorption mechanism (6), composite hydrogel (6), Graphene (6), methylene blue (6), wastewater treatment (6), dye removal (5), and gelatin (5). The scientific development in this period of years was led by the following authors: Wang Y., Wang X., Zhang Y., Liu Y., Wang J., Li J., Wang H., Chen Y., Li Y., and Li X. This period of years represented the production of most scientific articles about the principal themes of this work; meanwhile, the most recurring keywords referred to natural and thermosensitive hydrogel synthesis reinforced with graphene oxides for treatment of wastewater contaminated with dyes, as well as water purification.

### 3.2. Global Evolution of the Most Relevant Countries, Sources, and Authors

**Figure 3:**
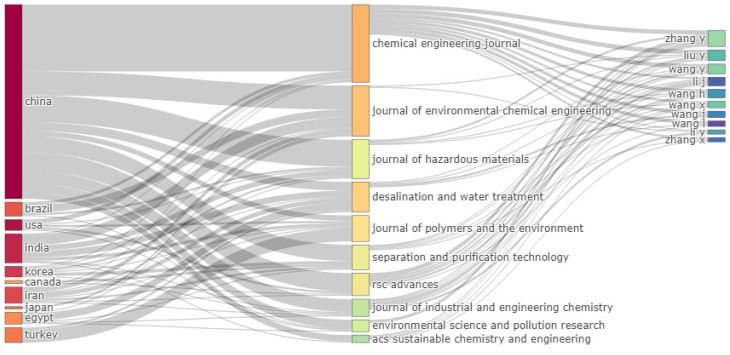
Three-field plot showing the network between countries (left), journals (middle), and authors (right) of original articles on the treatment of effluent industries with hydrogels from 2015 to 2022.

In Figure 3, a three-field diagram (Sankey diagram) was presented to qualitatively observe the scientific publication density and the relationship between countries (left), journals (middle), and authors (right) in reference to the publications during the period 2015–2022. The network width about the publication density and the network route establishes the relationship between country, journal, and author.

It was extracted from Figure 3 that the country that realized the most investigative production about hydrogel applications as adsorption materials of industrial contaminants was China, which produced almost 50% of published articles (570). Other relevant countries due to the productive mass of articles were India (61), Iran (49), USA (87), and Brazil (41). Likewise, the journals with the most density of published articles were the *Chemical Engineering Journal* (45), *Journal of Environmental Chemical Engineering* (30), and *Desalination and Water Treatment* (28). Concerning the h-index, the *Chemical Engineering Journal* had the most value with an $h_{index}=24$, namely, at least 24 published articles in this journal were cited 24 times; other relevant journals with high impact respect to the h index were: *Journal of Environmental Chemical Engineering* ($h_{index}=14$) and *Desalination and Water Treatment* ($h_{index}=9$).

On the other hand, in Figure 3, concerning the list of the most representative authors, Zhang Y. presented the highest number of produced articles (41), whose value of “articles fractionalized” was 7.2; additionally, the results regarding the authorship impact showed that Zhang Y. and Wang Y. had an $h_{index}$ equal to 17; however, Zhang Y. had a high score in to other impact indexes, such as the $g_{index}=41$, the total number of citations = 1811, and publication number = 41.

### 3.3. Thematic Evolution in the Period 2015–2022

**Figure 4:**
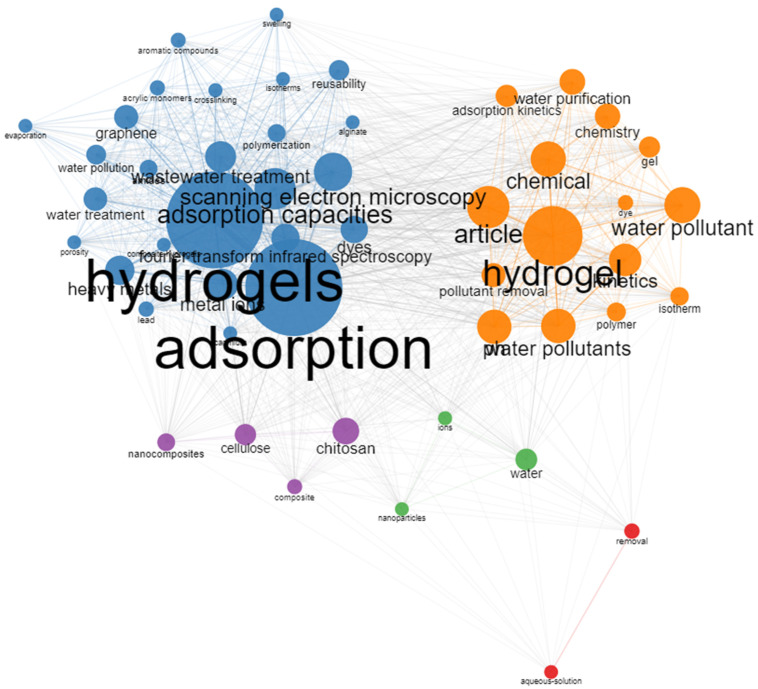
Keyword co-occurrence network about hydrogel applications on industrial effluents retrieved from the Scopus and Web of Science databases from 2015–2022. Note: Each node represents a different term, and each node diameter indicates the occurrence frequency.

Figure 4 represents the complete spectrum of the author keywords as a function of term occurrences in the articles. The label size is proportional to the keyword density and frequency. Furthermore, two big conglomerates of keywords joined through networks and located on the top of the bibliometric map were observed, which present a high interrelationship among keywords that make up each conglomerate. Meanwhile, these two high sets were relatively founded near each other; on the other hand, three clusters were observed on the bottom of the map separate and away from the two principal sets of keywords. Additionally, these three clusters showed less density and frequency of keywords.

Based on the sets obtained in the bibliometric map, five thematic groups were synthesized, which represent the principal thematic focus of the investigation:

Cluster N°1: “Adsorption hydrogels”. It includes the higher conglomerate of items whose keywords with higher density were hydrogels, adsorption, adsorption capacities, scanning electron microscopy, wastewater treatment, metal ions, and heavy metals.

Cluster N°2: “Hydrogels applied to the water treatment”. It represents the second higher conglomerate whose principal keywords were hydrogel, water pollutant, pollutant removal, water pollutants, water purification, article, adsorption kinetics, and chemical.

Cluster N°3: “Biodegradable hydrogels”. This group was made up of the following keywords: nanocomposites, cellulose, chitosan, and composite.

Cluster N°4: “adsorption nanoparticles”. It was made up of 3 keywords: ions, water, and nanoparticles.

Cluster N°5: “Aqueous contaminants removal”. It was formed by the removal of an aqueous solution.

**Figure 5:**
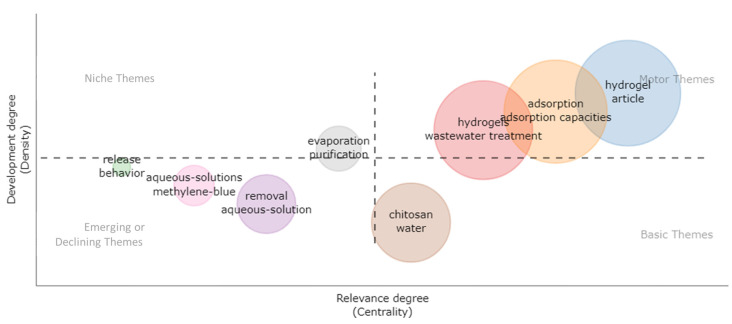
Thematic map of keywords regarding hydrogel applications in industrial effluents retrieved from the Scopus and Web of Science databases from 2015–2022. Note: Each node represents the keyword with the higher weight, and the diameter of each node means the density of the items.

Other visual tools regarding keywords and scientific study targeting generated the thematic map (Figure 5) in which four differentiated categories were defined according to the locate quadrant.

The three nodes located in the quadrant “Motor Themes” (high relevance degree and high development degree) were formed by “hydrogels and wastewater treatment”, “adsorption and adsorption capacities”, and “hydrogel and article”. This quadrant contained principal studies whose relevancy for the investigative front was of a specialized type; those studies were well-developed and important for scientific progress.

The nodes located in the quadrant “peripheral themes” (high relevance degree and high development degree) were formed by “evaporation and purification”. This quadrant contained peripheral studies whose investigation was according to the principal themes’ guidelines; however, their scientific production was limited.

The localized nodes in the lower left quadrant “Emerging or declining themes” (low relevance degree and low development degree) were mainly structured by “release and behavior”, “aqueous-solutions and methylene-blue”, and “removal and aqueous-solution”. The studies in this quadrant were considered emerging or declining, namely, they were in a consolidation phase; although much research has been done about those studies, a clear concept regarding those studies has not been made. Meanwhile, this quadrant was formed by studies whose theme was rejected because it was not important or relevant.

Finally, in the lower right quadrant, we found a node represented by the keywords “chitosan and water”; this quadrant was dominated by “basic and transversal themes” (high relevance degree and low development degree). The studies located in this section were those with high importance for a determined field of investigation, but they were not well developed by the scientific community.

In summary, the figure of the thematic map gives a first impression of the direction of the selected studies during the period 2015–2022. The adsorption through hydrogels to the wastewater treatment and contaminated water body are those themes to boost and are located on the investigation front; meanwhile, the referent works regarding colorant removal (methylene blue), ionic compounds, and contaminates present in aqueous solutions were founded in the consolidation phase because they were emerging studies whose scientific production was beginning to take shape. In addition, the purification and evaporation studies through porous membranes showed important information for the principal thematic development, but they still did not have a highly relevant production.

### 3.4. Systematic Analysis of the Selected Literature

Figure 1 shows the decision flow for selecting the competent articles for the study. First, two specialized databases in the science and engineering areas were identified: Scopus and Web of Science; 496 pertinent articles were obtained from the former and 460 articles from the latter. Then, the 956 articles were gathered and saved in BIB and CSV formats for their verification, refinement, and selection. Afterward, the documents were uploaded to the free web tool Rayyan for their manual and automatic revision of duplicates, selection of pertinent articles based on titles, and abstract revision of each article (1st filter). The inclusion or exclusion of duplicated articles was made through the following criteria: direct elimination (>90%), titles and principal indices revision (70%<%<90%) and exhaustive lecture of abstract (<70%). Afterward, the Excel 2016 tool was used as a second manual and automatic revision of duplicates; meanwhile, articles were selected based on title and abstract criteria (2nd filter). The results obtained were 125 articles in total for their later analysis based on eligibility criteria; meanwhile, 627 articles were excluded through titles and abstract revisions.

After the title and abstract analysis and before the analysis by eligibility criteria, 22 articles were excluded because they could not be retrieved for their exhaustive evaluation. Then, an eligibility analysis was done on the 103 remaining articles, of which 32 articles were excluded based on language criteria, 12 by not being inside the range of the established years for the present study, and 36 were excluded based on article content criteria (study scope, investigative thematic, study strictness, investigation design); thereby, a group of 23 articles was consolidated as the final sample.

Below is presented a synthesis chart of the selected articles, with their objectives and contributions regarding hydrogel applications as a technology tool for the treatment of industrial effluents.

## 4. Discussion

Based on what was exposed in the synthesis chart (Table 3), below are some of the most relevant studies regarding the application in real cases.

Zhou [56] presented in their investigation NH2-Starch/PAA hydrogel as adsorbents for heavy metal removal from industrial effluents of the Shuikoushan Smelting Plant, Hengyang, Hunan province, China. The adsorption process was done in a batch reactor to determine kinetic parameters and adsorption yield; likewise, two fixed-bed adsorption columns in series were done to evaluate practical hydrogels application; operation conditions are described in Table 3. Batch study results showed an adsorption capacity of 254 times dry hydrogel weight to the initial concentration of 180 mg/L Cd (II) and 1 g/L of adsorbent dose; on the other hand, the assays of actual effluents adsorption made in fixed-bed columns exhibited a relationship between adsorption percentage and adsorbent dose, so that to 4 g/L of adsorbent dose, the concentration of heavy metals reached lower values of 0.001 mg/L, except for Zn (21.95 mg/L) and TOC (14.35 mg/L).

Regarding bed efficacy, it was observed that the column can work continuously for a period of eight cycles reaching to volume treated of 2400 BV (27.1 L) and Cd (II) concentration was below 0.01 mg/L; each cycle finalization the second column took the first position, while the last put through a regeneration process. In this way, obtaining both columns in optimal conditions was achieved; in addition, a 1% advance in Cd (II) concentration in the breakthrough curve was observed after 228 BV. Column regeneration was made with a volume of 67.8 mL of HCl 0.1M solution, namely, when 2400 BV of wastewater was treated, 6 BV (67.8 mL) of eluent was necessarily used for column regeneration. Regarding the loss of hydrogel mass in the adsorption–regeneration process, the assays showed that loss achieved 1% of the total mass of the hydrogel, so that the sludge produced amount was insignificant.

On the way to accomplishing SDG and according to the agreement of sustainable development proposed in 2015 and started in 2016, biodegradable hydrogels accomplish an important role in eliminating environmental contaminants; therefore, in recent years, the utilization of monomers or polymers based on natural sources, such as cellulose, chitosan [77], guar gum, starch, and organic wastes, have been studied for their synthesis in hydrogels and subsequently, application in wastewater treatment [78,79,80].

According to what was exposed, Ma et al. [61] made synthesis studies of hydrogels based on waste cotton fabric (WCFs) dissolved into NaOH/urea to former a cellulose viscous paste with activated groups (Cellulose/PAM DNHs), likewise, this study made applicative essays to heavy metals adsorption (Cd (II), Cu (II), Pb (II), Zn (II) y Fe (II) from Minmetals Copper Industry located in Hengyang, Hunan province, China). Those studies were made in batch reactors and fixed-bed columns, and the last showed great advantages compared to other adsorption equipment, such as a low-cost index and relatively easy scaling from design and operation parameters. The studies showed high heavy metals adsorption efficacy of employing continuous adsorption columns for many operation cycles (Table 3); however, when outlet effluent concentrations in columns exceeded the permissible limits (0.1 mg/L Cd (II), 1.0 mg/L Cu (II), 1.0 mg/L Pb (II), 4.0 mg/L Zn (II), and 10 mg/L Fe (II)), column regeneration was carried out for its reutilization in the next cycle. To maintain the columns’ activity for a prolonged time, the study determined that treatment volume must be delimited to work below 42 BV (1932 mL); likewise, this work parameter allowed the elimination of the ions present in the solution in an efficacious way; the operation time achieved pH = 5.0 and T = 25 °C in 420 min. The elution of saturated columns was carried out by washing with 0.1 M HCL to a volume of 2 BV (92 mL).

Among the industries that emit the most pollutants through effluents and emissions, the mining–metallurgical industry is considered one of the main tailings and mineral sludge generators. Burillo et al. [59] conducted characterization studies of tailing and mining studies located in an acid mine drainage site near Arroyo San Pedro (San Luis Potosi, Mexico); likewise, a chitosan network (net-CS) was synthesized for Fe (III) and As (V) removal. The process was performed in batch reactors with an operation time of 50 h. The results showed that the present tailings were adsorbed satisfactorily, with a basic pH and environment temperature, by chitosan hydrogels (0.786 mg/g As (V) y 76.85 mg/g Fe (III)); in addition, the equilibrium studies (Freundlich isotherm) and kinetics studies (Pseudo-first order) manifested information regarding the adsorption nature, highlighting that the adsorption was a heterogeneous process governed by chemical adsorption.

According to the mentioned studies, Zhou et al. [60] worked on the adsorption of actual effluents from the Smelting Plant located in Hunan, China. The heavy metal removal from industrial effluents was carried out through fixed-bed columns packed with Jute/PAA hydrogels; likewise, equilibrium, kinetics, and the influence of principal variables, such as pH, initial concentration, initial dose, and temperature, were made. The obtained results showed that adsorption equilibrium was achieved in just 10 min using 1 g/L of adsorbent when contaminants were present at initial concentrations of 40 mg/L $Cd^{2+}$ and 40 mg/L $Pb^{2+}.$ The studies of fixed-bed columns revealed that effluents, after the adsorption process, contained a metallic ion concentration below 0.001 mg/L in an operation time of 2 h using 4 g/L of adsorbent. The regeneration of adsorption columns was carried out by 50 BV (565 mL); in other words, the adsorption columns were treated with 2900 BV (32.8 L) of industrial effluents, and 50 BV (565 mL) of eluent was needed to regenerate them.

Lin et al. [64] studied distillation membrane wetting for the treatment of wastewater of superficial low tension coming from the dyeing industry. This wetting was principally occasioned by surfactants (sodium dodecyl sulfonate (SDS), Tween20, and Tween85), which reduced the purification efficiency of the membranes. For this reason, a film of agarose hydrogel adhered to the Teflon membrane. The results showed that the distillation membrane did not present wetting during the first 24 h when the initial concentration of surfactant was 10 mg/L. Nevertheless, when the surfactant achieved a concentration above the critical micelle concentration (CMC), it was observed that surfactant penetration toward the membrane, at a slow pace and low way. The disadvantages observed in the anti-wetting distillation membrane system (Teflon membrane + agarose hydrogel film) were the operation flow, which was reduced by 70% when the hydrogel film was used; the temperature of operation, which showed a reduction of repulsion efficiency when it worked to high temperatures, this happened because the thermal stability of agarose hydrogels decreased when the operation temperature increased; furthermore, specifically in this study case, the membrane had a temperature gradient, which was the motor force of the operation ($T_{cold}=21\;{}^{\circ}C,T_{hot}=60\;{}^{\circ}C$). Thus, the application of this hydrogel is not recommended when it is worked to very high temperatures. On the other hand, micelle presence allowed the surfactant to penetrate through the hydrogel film and caused membrane wetting. A viable alternative regarding hydrogel selection could be the implementation of thermosensitive hydrogels, such as N-isopropylacrylamide (NIPAAm) and N-acryloylpyrrolidine [81].

Another important subject in this context is the global agreement about the management of the environment, specifically for this study, water management (SDG N°6), which mentions that it is necessary to guarantee water availability for all people, necessitating its sanitation and purification. According to that, He et al. [76] carried out studies about treatments for water bodies contaminated with fluorides $\left(F^{-}\right)$; a series of new hydrogels of Y-GO-SA were synthesized as adsorbents, and the studies were carried out in batch reactors, and fixed-bed columns. The results showed a high adsorption capacity (288.96 mg/g $F^{-}$) in 24 h of operation time; however, the adsorption presented efficiency problems of removal due to the presence of phosphate ions; this could be explained by the ionic charge of $PO_{4}^{3-}$ and $F^{-}$, which competed for the activated sites of the hydrogel internal structure; this selectivity depends on ionic molecule size (the smaller the ion size, the higher the ion adsorption will be by hydrogels).

On the other hand, the studies of adsorption columns provided high adsorption results (152.3 mg/g $F^{-}$), although they were lower than the studies made in batch reactors; furthermore, modeling studies were done to understand the adsorption dynamic, as a function of outlet solution from the fixed-bed column to determine the breakpoint, the breakthrough curve, and the exhaustion point; with that information, the adsorption efficacy as a function of time can be studied. Likewise, the breakthrough curve can determine the efficacy decrease of the column, namely, this curve is elicited by its regeneration time through washing with HCl. Concerning the fluoride adsorption, Thomas’s model fitted so well to experimental data that it had a continuous flow. Other applicative works have presented studies about adsorption process modeling in fixed-bed columns based on the Thomas model [55,65,69,70]; nevertheless, not all adsorption processes can be described by this model; for instance, the adsorption studies of ^137^Cs y ^134^Cs using PVA-alginate encapsulating Prussian blue-graphene oxide (PB-GO) hydrogel beads [62] showed that adsorption dynamics were fitted best by The Yoon–Nelson Mathematical Model and Thomas model. However, other important indicators such as Chi-square values ($\chi^{2}$) and average percentage errors (APE), which evaluate the fitness of the breakthrough model, showed that the Yoon–Nelson Mathematical Model (0.08–0.17 and 12.64–42.69) presents good agreement with the experimental data compared to the Thomas model (0.08–0.22 and 14.36–45.50). Other works, such as Song et al. [68], studied the adsorption of Malachite green through fixed-bed columns, and their results demonstrated that the kinetics process was fitted by the Adams–Bohart model [68].

The hydrogels as purification materials of water bodies have achieved important relevance due to their adsorption properties to eliminate contaminants, easy operation control, fast regeneration, reutilization, and low operative cost. These statements were supported by the study of Sharma and Tiwari [72], who presented the treatment of contaminated water bodies (Shankhini River, Dantewada, Chhattisgarh, India) through adsorption of $Fe^{2+}$ y $Fe^{3+}$ with nano-ZnO-loaded poly (acrylamide-co-itaconic acid) hydrogel (PAI). The study was done using fixed-bed adsorption columns and batch reactors. The removal efficacy in both systems achieved values upper to 99.0% of iron adsorption; likewise, the study evidenced that the columns could work in many cycles of adsorption–regeneration without manifesting a detectable loss of hydrogel efficacy. The design and operation parameters could be easily scaled to the removal of contaminants present in high wastewater volumes. On the other hand, the operation and capital costs were lower than the implementation of other removal technologies of contaminants, such as (1) membranes, (2) ozonation, and (3) electrodialysis; thus, practical and economic viability was evidenced for iron removal from rivers, underground water, or industrial effluents by PAI hydrogels.

## 5. Conclusions

The implementation of global goals to use, manage, and sanitize hydric resources has promoted the development of scientific studies about hydrogel utilization as adsorption materials for the treatment of industrial effluents; as a consequence, the production of scientific articles regarding the removal of industrial contaminants through batch reactors and fixed-bed adsorption columns has increased in recent years. Likewise, the easy operational control of equipment, low operational cost, low maintained cost, and straightforward scaling for the treatment of high volumes of wastewater have been fundamental to determine the viability of this new treatment methodology of industrial effluents. 

The present study allows visualization of the current state of hydrogel implementation in the industry, and its advantages and disadvantages against diverse operation conditions. Likewise, the synthesis chart allows us to obtain design and operation data about unit equipment used in the adsorption of industrial contaminants. 

On the other hand, it was mentioned that the study scope limited the information searching to other languages, such as Chinese, since the bibliometric and systematic studies showed that China is the country where the most scientific advances have been developed regarding hydrogel application in the treatment of actual industrial effluents.

## Figures and Tables

**Table 1:** Comparison of different methods to eliminate contaminants present in wastewater.

Technology	Principle	Disadvantages
Catalytic ozonation [16]	Advanced oxidation	Short useful life of ozone, high energetic requirement, and sample contamination
Membrane filtration [4]	Physic separation	Short useful life of membranes, membrane soiling, and no membrane regeneration
Microwave catalysis [21]	Energetic decomposition	High investment costs and lack of secondary studies
Photocatalytic degradation [22]	Catalysis by ultraviolet light and catalysts of TiO_2_ (titanium dioxide) and ZnO (zinc oxide)	No applicability to the industrial level
Coagulation-flocculation [24]	Separation by conglomerates	Use of toxic chemicals and generation of sludge
Chemical oxidation [4]	Rupture of chemical bonds	High energetic demand
Electrochemical degradation [20]	Electrochemical reaction	Low efficiency and high operational costs
Electro- flocculation [4]	Conglomerate of contaminants through electric current	No applicability to the industrial level and high energetic requirement
Ion exchange [24]	Ion exchange between a solid and liquid without mass transfer	Soiling, costs in adsorbent regeneration, costs in adsorbent reactivation, maintenance costs
Chemical Precipitation [4]	Transformation through chemical reactions, evaporation, cooling, and so on, of dissolved compounds to a precipitable solid	Formation of large amounts of sludge
Adsorption [18]	Concentration of one or more components of a solute in an internal or external surface of a solid adsorbent	Performance depends on the adsorbent material selection, maintenance, and regeneration costs

Note: Own elaboration.

**Table 2:** The sequence of search algorithms in the Scopus and Web of Science databases.

Databases	Searching Algorithm
Scopus	TITLE (hydrogel* AND (water OR treatment OR tailing* OR effluent* OR adsorption)) AND NOT (drug OR cancer OR tumor OR delivery)
Web of Science	WC = (engineering) AND TI = (hydrogel OR hydrogels) AND TI = (water OR treatment OR tailing OR effluent OR adsorption) NOT TI = (drug OR tumor OR cancer) NOT AB = (drug OR tumor OR cancer) NOT AK = (drug OR tumor OR cancer)

Note: Own elaboration.

**Table 3:** Synthesis of included studies’ content of the systematic review process.

Industrial Effluent Treatment										
Author	Hydrogel (Adsorbent)	Sector	Objective	Equipment	Parameters				Contribution	Source
					Removal Efficacy	Operation Conditions	Adsorption Isotherm	Adsorption Kinetics		
Jain et al.	polyvinyl alcohol-glutaraldehyde cross-linked hydrogel beads (PVA/GA)	Textile Industry	Removal of industrial colorant Congo Red (CR)	Fixed-bed column	25.9 mg/g CR	H * (cm) = 60	Thomas model		PVA/GA hydrogel beads were synthesized; it was low cost and able to remove CR dye completely. The operation time was around 1–2 h. This process showed reutilization cycles, which could have been the seventh time. In addition, Thomas’s model perfectly described the fixed-bed breakthrough curve, where the internal and external mass diffusion did not limit the adsorption velocity.	[55]
						F (mL/min.) = 20				
						pH = 6.0				
						T (°C) = 25				
				Batch reactor	34 mg/g CR	pH = 6.0	Harkins Jura	Pseudo-second order		
						T (°C) = 45				
Zhou et al.	amino-functionalized Starch/PAA hydrogel (NH2-Starch/PAA)	Metallurgical Industry	Heavy metal and organic carbon (TOC) removal from Shuikoushan Smelting Plant, Hengyang, Hunan province, China	Fixed-bed column	94.3% Pb, 93.1% Cd, 71.6% Mn,	H (cm) = 10			The synthesis of NH2-starch/PAA hydrogel for the elimination of the heavy metals from wastewater showed applicability in a large pH range, getting the equilibrium steady very quickly. Furthermore, the adsorbent showed clear advantages (high yield, easy regeneration, fast adsorption) concerning granular adsorbents and nano-adsorbents.	[56]
						F (mL/min.) = 2.26				
					98.5% Ni, 93.5% Cu, 99% Cr y 99% TOC	pH = 5.0				
						T (°C) = 25				
				Batch reactor	256.4 mg/g Cd (II)	pH = 5.0	Langmuir (Cd (II))	Pseudo-first order to low concentrations and Pseudo-second order to high concentrations (Cd (II))		
						T (°C) = 25				
Zhou, Luo, et al.	polyampholyte hydrogel strengthened with graphene oxide	Mining and metallurgical Industry	Heavy metal removal from Shuikoushan Smelting Plant, Hengyang, Hunan Province, China	Fixed-bed column	99.9% Cd, 91.3% Zn, 44.1% Mn,	H (cm) = 10			The design and synthesis of polyampholyte hydrogel strengthened with graphene oxide was done through polymerization with free radicals for the metallic ions’ adsorption from practical wastewater. The mechanical characteristics of hydrogels give them properties such as easy regeneration and reutilization. Additionally, the results showed a great removal capacity of heavy metals from industrial effluents.	[57]
						F (mL/min.) = 2.26				
					50.2% Ni, 31.0% Cu	pH = 5.0				
						T (°C) = 25				
				Batch reactor	216.1 mg/g Pb (II) y 15.8 mg/g Cd (II)	pH = 5.0 Pb (II) y 6.0 Cd (II)	Langmuir	Pseudo-second order		
						T (°C) = 40				
Peñas et al.	Cyclodextrin-Based	Manufacturing Industry	Cresols removal from industrial effluents	Fixed-bed column	6.2 mg/g (o-cresol), 11.6 mg/g (m-cresol) and 15.1 mg/g (p-cresol)	H (cm) = 7.5	Dose–response model for the sorption step and a pulse-peak model for the regeneration step		Fixed-bed columns based on CDP hydrogels has been demonstrated to be effective for organic compound removal present on industrial effluents; it showed reutilization capacity and structural stability. The Cyclodextrin matrix was effective in the explicit elimination of cresols present in water; it also showed high yield and reutilization capacity through regeneration with methanol.	[58]
						F (mL/min.) = 2.60				
						pH = 6.6				
	Hydrogels (β-CDP)					T (°C) = 25				
				Batch reactor	5.51 mg/g phenol	pH = 6.6	Langmuir and Freundlich	-		
						T (°C) = 25				
Burillo et al.	chitosan network (net-CS) and chitosan network-N-vinylcaprolactam/N–N-dimethylacrylamide (netCS)-g-NVCL/DMAAm hydrogels	Mining Industry	Leached effluents (As (V) and Fe (III)) removal from the mining district of San Luis Potosi, Mexico	Batch reactor	0.786 mg/g As (V) y 76.85 mg/g Fe (III)	pH = 2.9	Freundlich	Pseudo-first order	Chitosan network (net-CS) hydrogels showed a high removal capacity of As and Fe from mining tailing, so that, this hydrogel is technically and economically preferable as removal materials by its low cost and easy operatively. Freundlich isotherm perfectly described the adsorption of chitosan network (net-CS) hydrogels; this model mentioned that the process showed heterogeneous adsorption; likewise, the pseudo-first order model suggested that the process was done by chemical adsorption.	[59]
						T (°C) = 25				
Zhou et al.	double network Jute/Polyacrylic acid (Jute/PAA)	Metallurgical Industry	Heavy metals (Cd^2+^, Pb^2+^, Mg^2+^, Ca^2+^, K^+^, Na^+^, Cu^2+^, Zn^2+^, Mn^2+^) removal from Shuikoushan Smelting Plant, Hengyang, Hunan province, China	Fixed-bed column	81.0% Pb, 79.3% Cd, 83.4% Cu, 29.8% Zn, 22.3% Mn, 96.2% Cr, 99.8% Fe	H (cm) = 10			The Jute/PAA hydrogels synthesized showed great adsorption kinetic, well reutilization, and lower cost of raw materials. Jute/PAA hydrogels turned out to be effective in heavy metal removal of actual effluents coming from Shuikoushan Smelting Plant, Hengyang, Hunan province, China, achieving values below 0.001 mg/L in a time operation of 2 h.	[60]
						F (mL/min.) = 2.26				
						pH = 5.0				
						T (°C) = 25				
				Batch reactor	401.7 mg/g de Cd^2+^ y 542.9 mg/g de Pb^2+^	pH = 5.0	Langmuir	Pseudo-first order		
						T (°C) = 25				
Ma et al.	double network hydrogel (Cellulose/PAM DNHs)	Mining-metallic Industry	Heavy metals (Cd (II), Cu (II), Pb (II), Zn (II) y Fe (II)) removal from Minmetals	Fixed-bed column	99.8% Cd (II), 99.8% Cu (II), 99.7% Pb (II),	H (cm) = 15			Waste cotton fabrics (WCF) reutilization to the synthesis of low-cost double network (Cellulose/PAM DNHs) hydrogels to heavy metals elimination from the Minmetals Copper Industry located in Hengyang, Hunan province, China. These hydrogels present a way of waste cotton valorization; likewise, heavy metal removal from industrial wastewater contributes to reducing pollutants from the environment and boosting the sustainability of natural resources.	[61]
						F (mL/min.) = 5.75				
					99.9% Zn (II), 98.0% Fe (II)	pH = 5.0				
			The copper industry located in Hengyang, Hunan Province, China			T (°C) = 25				
				Batch reactor	198,48 mg/g Cd (II), 138,90 mg/g Cu (II), 382,80 mg/g Pb (II),	pH = 5.0	Langmuir	Pseudo-second order		
						T (°C) = 25				
Jang y Lee	PVA-alginate encapsulated Prussian blue graphene oxide (PB-GO) hydrogel beads	Nuclear Industry	^137^Cs and ^134^Cs removal from a nuclear plant	Fixed-bed column	164.5 mg/g	H (cm) = 20	Yoon-Nelson Mathematical Model		PB-GO hydrogel beads could remove Cs contaminants from nuclear plant effluents, and they are developed in fixed-bed columns making them easy to scale for industrial applications. The Yoon–Nelson model correctly described the breakthrough curve of a fixed-bed column packed with PB-GO hydrogels.	[62]
						F (mL/min.) = 0.83				
						pH = 7.0				
						T (°C) = 20				
Sharma y Tiwari	nanomagnetite-loaded poly (Acrylamide-co-itaconic acid) hydrogel (PAI)	Mining and Metallurgical Industry	Mn (II) removal from industrial effluents	Fixed-bed column	99.55 % Mn (II)	H (cm) = 10			The nano magnetite-loaded poly (Acrylamide-co-itaconic acid) hydrogels (PAI) can be reutilized many times without present deficiency in adsorption capacity. Meanwhile, fixed-bed column parameters can be scaled easily to remove industrial effluents of high volume.	[63]
						F (mL/min.) = 1				
						pH = 6.0				
						T (°C) = 25				
				Batch reactor	99.04% Mn (II)	pH = 6.0	Freundlich	Pseudo-second order		
						T (°C) = 25				
Sezgin y Balkaya	polyacrylic acid (Aac) hydrogel	Galvanotechnic Industry	Cu (II), Ni (II), Zn (II), and total Cr from galvanotechnic industrial site in Istanbul	Batch reactor	2.74 mg/g Cu (II),1.91 mg/g Ni (II), 6.83 mg/g Zn (II) y 6.61 mg/g total Cr	pH = acido	Freundlich	Pseudo-second order	Polyacrylic acid (Aac) hydrogel showed to be a great adsorbent of heavy metals from the galvanotechnic industry. Likewise, the adsorption process showed good economic characteristics, high efficacy, and low operation time for ionic contaminants elimination.	[24]
						T = 20–50 °C				
Lin et al.	Agarose hydrogel	Dyeing Industry	Suppression of surfactant wetting during the membrane distillation process to the wastewater industrial treatment	Membrane distillation		Membrane dimensions = 20 × 20 cm^2^			The results showed the capacity of agarose hydrogels to eliminate surfactant wetting towards the distillation membranes during the treatment of dyes effluents. However, the raise in temperature detriments the efficacy of agarose hydrogels; it is recommended that the agarose hydrogel be changed to other ones, such as thermosensitive hydrogels.	[64]
						F (L/min.) = 2				
						pH = 6.0				
						Tcold (°C) = 21, Thot (°C) = 60				
Wan et al.	poly N-isopropylacrylamide/aluminum alginate (PNIPAM/AA) IPN hydrogel beads	Agrochemical Industry	PO_4_^−3^ removal from wastewater	Fixed-bed column	77.0% PO_4_^−3^	H (cm) = 30	Thomas model		The results showed PNIPAM/AA hydrogels had a strong capacity for phosphate adsorption and the studies made in fixed-bed columns to scale to the industrial level (large effluent volumes) in an easy way. The column adsorption showed a great removal of PO4-3 in 180 min of operation time.	[65]
						F (mL/min.) = 6				
						pH = 3.0				
						T (°C) = 35				
				Batch reactor	16.51 mg/g PO_4_^−3^	pH = 3.0	Freundlich y Slips	Pseudo-second order and intraparticle diffusion		
						T (°C) = 35				
Yi et al.	three-dimensional (3D) cobalt hydroxide (CoOOH)/graphene oxide (GO) hydrogel	Farming and Agrochemical Industry	Removal of organic pollutants from industrial wastewater	Degradation column	95%	H (cm) = 10	-	-	The degradation column system incorporated with CoOOH/GO hydrogels demonstrated excellent catalytic activity for the different organic pollutants; it also provided geometric information and operating conditions for its application on an industrial scale.	[66]
						F (mL/min.) = 200				
						pH = 6.0				
						∆P (Pa) = 2300				
He et al.	cross-linked sodium acrylate and acrylamide copolymer/graphene oxide (P (AANa-co-AM)/GO) hydrogels	Mining and Metallurgical Industry	Removal of heavy metals Pb^2+^ and Cd^2+^	Fixed-bed column	87.5% Pb^2+^	H (cm) = 10			The adsorption through hydrogels can simultaneously eliminate different heavy metals from industrial wastewater. Likewise, its efficacy was not prejudiced after 5 cycles of adsorption-regeneration. Thus, it was concluded GO/P (AANa-co-AM) hydrogels present a great operational yield in the treatment of industrial effluents.	[67]
						F (mL/min.) = 2				
						pH = 4.5 Pb^2+^				
						T (°C) = 25				
				Batch reactor	452.3 mg/g Pb^2+^ and 196.4 mg/g Cd^2+^	pH = 4.5 Pb^2+^ y 6.0 Cd^2+^ T (°C) = 25	Langmuir	Pseudo-first orden		
Song et al.	Chitosan hydrogel beads	Paper and Textile Industry	Malachite green (MG) oxalate removal from industrial effluents	Fixed-bed column	≈100% MG	H (cm) = 25	Modified Adams-Bohart model		Fixed-bed columns based on chitosan hydrogel beads demonstrated high malachite green (MG) adsorption from industrial effluents; the operation time was around 3 h, which is a great candidate for its scaling to an industrial level. Furthermore, the studies done on fixed-bed columns regarding adsorption kinetics were explained by a modified Adams–Bohart model due to the complexity of mass transfer mechanisms.	[68]
						F (mL/min.) = 0.97–4.95				
						pH = 7.5				
						T (°C) = 25				
				Batch reactor	91% MG	pH = 7.5	Langmuir	Pseudo-second order		
						T = 25 °C				
Yan et al. (2020)	nanoCaCO3 in-situ encapsulated hydrogels (CCx-CB) carbonaceous beads	Farming and Pharmaceutical Industry	Tetracycline (TC) removal from industrial effluent	Fixed-bed column	69.6 mg/g TC	H (cm) = 10	Thomas model		The results showed technical and economic viability regarding the utilization of CCx-CB hydrogels in fixed-bed columns for TC removal. Furthermore, economic results demonstrated a cost reduction when raw materials of industrial degree were utilized in the hydrogel preparations.	[69]
						F (mL/min.) = 1.00				
						pH = 7.0				
						T (°C) = 20				
				Batch reactor	279.3 mg/g TC	pH = 7.0	Langmuir	Pseudo-second order		
						T (°C) = 20				
Pei et al.	Porous alginate-based hydrogel beads (porous ABH)	Paper and textile Industry	Methylene blue (MB) removal from textile industrial effluents	Fixed-bed column	1907.76 mg/g MB	H (cm) = 12	Thomas model		The study results showed great removal capacity for MB adsorption from industrial effluents using ABH-1:3 hydrogels. Furthermore, the removal percentage remained up to 75%, even after 10 reutilization cycles. Likewise, ABH-1:3 hydrogels demonstrated efficiency and efficacy in textile industrial contaminant removal.	[70]
						F (mL/min.) = 5				
						T (°C) = 25				
				Batch reactor	1320 mg/g MB	pH = 2.0–12.0	Langmuir	Pseudo-second order		
						T (°C) = 25				
Bethi et al.	Halloysite nanoclay embedded poly-acrylic acid (PAA) nanocomposite hydrogel	Textile Industry	Rhodamine (Rh-B) removal from textile industrial effluents	Hybrid system (Hydrodynamic cavitation + adsorption)	65% Rh-B	Masa de hidrogel cargada (g) = 25			Rh-B removal was around 65% through a hybrid hydrodynamic cavitation system and adsorption using halloysite nanoclay embedded poly-acrylic acid (PAA) nanocomposite hydrogel; in addition, it was found that efficacy rose to 72.85% when H_2_O_2_ was added to the hybrid system.	[71]
						pH = 7.62				
						Tiempo de operación (min) = 120				
**Depuration and Purification Technology**										
**Author**	**Hydrogel (Adsorbent)**	**Sector**	**Objective**	**Equipment**	**Parameters**				**Contribution**	**Source**
					**Removal Efficacy**	**Operation Conditions**	**Adsorption Isotherm**	**Adsorption Kinetics**		
Sharma y Tiwari	nano-ZnO-loaded poly (acrylamide-co-itaconic acid) hydrogel (PAI)	Municipal and Industrial Contaminants in Water Bodies	Fe^2+^ and Fe^3+^ removal from Shankhini River (Dantewada, Chhattisgarh, India)	Fixed-bed column	99.86% iron	H (cm) = 2			Nano-ZnO-loaded poly (acrylamide-co-itaconic acid) hydrogel (PAI) showed the elimination capacity of iron from Shankhini River to have minimum capital cost and great efficacy. Likewise, its column parameters make scaling easy to treat high volumes of industrial effluents.	[72]
						F (mL/min.) = 1.00				
						pH = 4.0				
						T (°C) = 25				
				Batch reactor	99.50% iron	pH = 4.0	Freundlich	Pseudo-second order		
						T (°C) = 25				
Zhuang et al.	porous graphene (GO)/alginate double network nanocomposite beads (GAD)	Textile Contaminants in Water Bodies	Methylene blue (MB) removal from contaminated water	Fixed-bed column	60.2% MB	H (cm) = 10			The studies demonstrated the removal capacity of methylene blue (MB) through GA hydrogels in the water purification process. Furthermore, GO addition to the adsorbent structure allowed hydrogel production to be large scale and low-cost, which gives them economic and technical viability for industrial scale applications.	[73]
						F (mL/min.) = 0.50				
						pH = 8.0				
						T (°C) = 25				
				Batch reactor	2.30 mg/g MB	pH = 8.0	Freundlich	Pseudo-second order		
						T (°C) = 25				
Gonçalves et al.	poly (acrylamide-co-sodium acrylate) hydrogels (pAAm-co-SA)	Fuel and Biofuel Industry	Water adsorption from biofuels	sieve plate column	94.1% water	H (cm) = NDA ***			Based on the results, it was concluded that the complete elimination of water from biodiesel was achieved. Likewise, bed configuration presents great advantages due to the geometry of its plates, which could eliminate water content from different fuels without any worries about bed clogging.	[74]
						F (mL/min.) = 5.00				
						T (°C) = 25				
						pH = NDA				
S. Dong et al.	layered double hydroxides-isethionate (LDH-ise) assisted covalent and electrostatic crosslinked cationic hydrogel (CH-LDH-ise)	Drinking and Underground Water Treatment	Remoción de Cr (VI) removal from aqueous solutions	Fixed-bed column	≈100%	H (cm) = 10			Adsorption efficacy of CH-LDH-ise hydrogels to Cr (VI) removal from water bodies and its reutilization capacity through fixed-bed column regeneration give it favorable characteristics of efficiency and fast ionic contaminants elimination from industrial effluents. In addition, an efficacy of nearly 100% Cr (VI) was observed through a treatment volume of 2250 bed volumes ** (BV).	[75]
						F (mL/min.) = NDA				
						pH = NDA				
						T (°C) = 30				
				Batch reactor	408.4 mg/g Cr (VI)	pH = 7.0	Sips	Pseudo-second order		
						T (°C) = 30				
He et al.	A novel 3D yttrium based-graphene oxide-sodium alginate hydrogel	Drinking Water Treatment	Water body purification through fluorides purification (F^−^)	Fixed-bed column	152.3 mg/g F^−^	H (cm) = 4.5	Thomas model		The obtained results showed high removal efficacy of fluorides from water bodies using Y-GO-SA hydrogels in batch and fixed-bed column processes Likewise, batch studies demonstrated more efficacy than adsorption studies of fixed-bed columns. On the other hand, adsorption columns presented a reutilization capacity through regeneration cycles, which is essential in water potabilization.	[76]
						F (mL/min.) = 0.2–0.5				
						pH = 6.5				
						T (°C) = 20				
				Batch reactor	288.96 mg/g F^−^	pH = 4.0	Langmuir	Pseudo-first order and Pseudo-second order		
						T (°C) = 20				

* H: Bed height. ** bed volumes = Bed height (cm) × Column cross-sectional area (cm^2^)/1000. *** NDA: No data available.

## Data Availability

Not applicable.

## References

[1] Schweitzer L., Noblet J. (2017). Chapter 3.6—Water contamination and pollution. Green Chemistry: An Inclusive Approach.

[2] Wang Q., Yang Z. (2016). Industrial water pollution, water environment treatment, and health risks in China. Environ. Pollut..

[3] Ilyas M., Ahmad W., Khan H., Yousaf S., Yasir M., Khan A. (2019). Environmental and health impacts of industrial wastewater effluents in Pakistan: A review. Rev. Environ. Health.

[4] Crini G., Lichtfouse E. (2018). Advantages and disadvantages of techniques used for wastewater treatment. Environ. Chem. Lett..

[5] Wang Q., Wang X., Liu Y., Li R. (2021). Urbanization and water consumption at national- and subnational-scale: The roles of structural changes in economy, population, and resources. Sustain. Cities Soc..

[6] Liu J., Wang Y., Yu Z., Cao X., Tian L., Sun S., Wu P. (2017). A comprehensive analysis of blue water scarcity from the production, consumption, and water transfer perspectives. Ecol. Indic..

[7] Rehman A., Chandio A.A., Hussain I., Luan J. (2019). Fertilizer consumption, water availability and credit distribution: Major factors affecting agricultural productivity in Pakistan. J. Saudi Soc. Agric. Sci..

[8] Kaika M. (2003). The Water Framework Directive: A New Directive for a Changing Social, Political and Economic European Framework. Eur. Plan. Stud..

[9] Rabadán A., Sáez-Martínez F. (2017). Why european entrepreneurs in the water and waste management sector are willing to go beyond environmental legislation. Water.

[10] Kochskämper E., Challies E., Newig J., Jager N.W. (2016). Participation for effective environmental governance? Evidence from Water Framework Directive implementation in Germany, Spain and the United Kingdom. J. Environ. Manag..

[11] Giupponi C., Gain A.K. (2017). Integrated water resources management (IWRM) for climate change adaptation. Reg. Environ. Chang..

[12] Bebbington J., Unerman J. (2018). Achieving the United Nations Sustainable Development Goals. Account. Audit. Account. J..

[13] Priya Sharma A.K., Kaith B.S., Tanwar V., Bhatia J.K., Sharma N., Bajaj S., Panchal S. (2019). RSM-CCD optimized sodium alginate/gelatin based ZnS-nanocomposite hydrogel for the effective removal of biebrich scarlet and crystal violet dyes. Int. J. Biol. Macromol..

[14] Ertugay N., Acar F.N. (2017). Removal of COD and color from direct blue 71 azo dye wastewater by fenton’s oxidation: Kinetic study. Arab. J. Chem..

[15] Piaskowski K., Świderska-Dąbrowska R., Zarzycki P.K. (2018). Dye Removal from Water and Wastewater Using Various Physical, Chemical, and Biological Processes. J. AOAC Int..

[16] Wang J., Chen H. (2020). Catalytic ozonation for water and wastewater treatment: Recent advances and perspective. Sci. Total Environ..

[17] Kıvanç M.R., Yönten V. (2020). A statistical optimization of methylene blue removal from aqueous solutions by Agaricus Campestris using multi-step experimental design with response surface methodology: Isotherm, kinetic and thermodynamic studies. Surf. Interfaces.

[18] Li T., Liu L., Zhang Z., Han Z. (2020). Preparation of nanofibrous metal-organic framework filter for rapid adsorption and selective separation of cationic dye from aqueous solution. Sep. Purif. Technol..

[19] Bento R.M., Almeida M.R., Bharmoria P., Freire M.G., Tavares A.P. (2020). Improvements in the enzymatic degradation of textile dyes using ionic-liquid-based surfactants. Sep. Purif. Technol..

[20] Ozturk D., Yilmaz A.E. (2020). Investigation of electrochemical degradation of Basic Red 13 dye in aqueous solutions based on COD removal: Numerical optimization approach. Int. J. Environ. Sci. Technol..

[21] Zuo S., Li D., Xu H., Xia D. (2020). An integrated microwave-ultraviolet catalysis process of four peroxides for wastewater treatment: Free radical generation rate and mechanism. Chem. Eng. J..

[22] Chen J., Zhang X., Shi X., Bi F., Yang Y., Wang Y. (2020). Synergistic effects of octahedral TiO2-MIL-101(Cr) with two heterojunctions for enhancing visible-light photocatalytic degradation of liquid tetracycline and gaseous toluene. J. Colloid Interface Sci..

[23] Bustos-Terrones Y.A., Hermosillo-Nevárez J.J., Ramírez-Pereda B., Vaca M., Rangel-Peraza J.G., Bustos-Terrones V., Rojas-Valencia M.N. (2021). Removal of BB9 textile dye by biological, physical, chemical, and electrochemical treatments. J. Taiwan Inst. Chem. Eng..

[24] Sezgin N., Balkaya N. (2015). Adsorption of heavy metals from industrial wastewater by using polyacrylic acid hydrogel. Desalin. Water Treat..

[25] Pereira A., Martins A., Paulino A., Fajardo A., Guilherme M., Faria M., Linde G., Rubira A., Muniz E. (2017). Recent Advances in Designing Hydrogels from Chitin and Chitin-Derivatives and their Impact on Environment and Agriculture: A Review. Rev. Virtual Quim..

[26] Tran V., Park D., Lee Y. (2018). Hydrogel applications for adsorption of contaminants in water and wastewater treatment. Environ. Sci. Pollut. Res..

[27] Samaddar P., Kumar S., Kim K.H. (2019). Polymer hydrogels and their applications toward sorptive removal of potential aqueous pollutants. Polym. Rev. (Phila Pa).

[28] Verma A., Thakur S., Mamba G., Prateek Gupta R., Thakur P., Thakur V. (2020). Graphite modified sodium alginate hydrogel composite for efficient removal of malachite green dye. Int. J. Biol. Macromol..

[29] Santos C.M.D.C., Pimenta C.A.D.M., Nobre M.R.C. (2007). The PICO strategy for the research question construction and evidence search. Rev. Lat. Am. Enferm..

[30] Pan Y., Yin C., Fernandez C., Fu L., Lin C.T. (2022). A Systematic Review and Bibliometric Analysis of Flame-Retardant Rigid Polyurethane Foam from 1963 to 2021. Polymers.

[31] Olisah C., Okoh O.O., Okoh A.I. (2018). A bibliometric analysis of investigations of polybrominated diphenyl ethers (PBDEs) in biological and environmental matrices from 1992–2018. Heliyon.

[32] Reis C.H.B., Buchaim D.V., Ortiz A.D.C., Fideles S.O.M., Dias J.A., Miglino M.A., Teixeira D.D.B., Pereira E.D.S.B.M., da Cunha M.R., Buchaim R.L. (2022). Application of Fibrin Associated with Photobiomodulation as a Promising Strategy to Improve Regeneration in Tissue Engineering: A Systematic Review. Polymers.

[33] Zhu J., Liu W. (2020). A tale of two databases: The use of Web of Science and Scopus in academic papers. Scientometrics.

[34] Zyoud S.H., Waring W.S., Al-Jabi S.W., Sweileh W.M. (2017). Global cocaine intoxication research trends during 1975–2015: A bibliometric analysis of Web of Science publications. Subst. Abus. Treat. Prev. Policy.

[35] Rovira C., Codina L., Guerrero-Solé F., Lopezosa C. (2019). Ranking by Relevance and Citation Counts, a Comparative Study: Google Scholar, Microsoft Academic, WoS and Scopus. Future Internet.

[36] Aria M., Cuccurullo C. (2017). Bibliometrix: An R-tool for comprehensive science mapping analysis. J. Informetr..

[37] R Core Team R: A Language and Environment for Statistical Computing (4.2.1). https://www.r-project.org.

[38] Haddaway N.R., Page M.J., Pritchard C.C., McGuinness L.A. (2022). *PRISMA 2020*: An R package and Shiny app for producing PRISMA 2020-compliant flow diagrams, with interactivity for optimised digital transparency and Open Synthesis. Campbell Syst. Rev..

[39] Ouzzani M., Hammady H., Fedorowicz Z., Elmagarmid A. (2016). Rayyan—A web and mobile app for systematic reviews. Syst. Rev..

[40] Vrabel M. (2015). Preferred Reporting Items for Systematic Reviews and Meta-Analyses. Oncol. Nurs. Forum.

[41] Moher D., Thombs B.D., McGrath T.A., Bossuyt P.M., Clifford T., Cohen J.F., Deeks J.J., Gatsonis C., Hooft L., Hunt H.A. (2018). Preferred Reporting Items for a Systematic Review and Meta-analysis of Diagnostic Test Accuracy Studies. JAMA.

[42] Moher D., Liberati A., Tetzlaff J., Altman D.G., The PRISMA Group (2014). Ítems de referencia para publicar Revisiones Sistemáticas y Metaanálisis: La Declaración PRISMA. Rev. Esp. Nutr. Hum. Diet..

[43] UNESCO and Sustainable Development Goals. https://en.unesco.org/sustainabledevelopmentgoals.

[44] Sánchez L.E., Croal P. (2012). Environmental impact assessment, from Rio-92 to Rio+20 and beyond. Ambient Soc..

[45] Lupi F., Gentile L., Gabriele D., Mazzulla S., Baldino N., de Cindio B. (2015). Olive oil and hyperthermal water bigels for cosmetic uses. J. Colloid Interface Sci..

[46] Bashari A., Shirvan A., Shakeri M. (2018). Cellulose-based hydrogels for personal care products. Polym. Adv. Technol..

[47] Farjami T., Madadlou A. (2019). An overview on preparation of emulsion-filled gels and emulsion particulate gels. Trends Food Sci. Technol..

[48] Li J., Jia X., Yin L. (2021). Hydrogel: Diversity of Structures and Applications in Food Science. Food Rev. Int..

[49] Caliari S.R., Burdick J.A. (2016). A practical guide to hydrogels for cell culture. Nat. Methods.

[50] Chaudhuri O. (2017). Viscoelastic hydrogels for 3D cell culture. Biomater. Sci..

[51] Skrzypczak D., Mikula K., Kossińska N., Widera B., Warchoł J., Moustakas K., Chojnacka K., Witek-Krowiak A. (2020). Biodegradable hydrogel materials for water storage in agriculture—Review of recent research. Desalin. Water Treat..

[52] Qu B., Luo Y. (2020). Chitosan-based hydrogel beads: Preparations, modifications and applications in food and agriculture sectors—A review. Int. J. Biol. Macromol..

[53] Fu L., Yu A., Lai G. (2021). Conductive Hydrogel-Based Electrochemical Sensor: A Soft Platform for Capturing Analyte. Chemosensors.

[54] Charaya H., La T., Rieger J., Chung H. (2019). Thermochromic and piezocapacitive flexible sensor array by combining composite elastomer dielectrics and transparent ionic hydrogel electrodes. Adv. Mater. Technol..

[55] Jain P., Sahoo K., Mahiya L., Ojha H., Trivedi H., Parmar A.S., Kumar M. (2021). Color removal from model dye effluent using PVA-GA hydrogel beads. J. Environ. Manag..

[56] Zhou G., Liu C., Chu L., Tang Y., Luo S. (2016). Rapid and efficient treatment of wastewater with high-concentration heavy metals using a new type of hydrogel-based adsorption process. Bioresour. Technol..

[57] Zhou G., Luo J., Liu C., Chu L., Ma J., Tang Y., Zeng Z., Luo S. (2016). A highly efficient polyampholyte hydrogel sorbent based fixed-bed process for heavy metal removal in actual industrial effluent. Water Res..

[58] Peñas F.J., Romo A., Isasi J.R. (2021). Removal of cresols from water by packed beds of cyclodextrin-based hydrogels. J. Polym. Environ..

[59] Burillo J.C., Castro-Larragoitia J., Burillo G., Ortega A., Medellin-Castillo N. (2017). Removal of arsenic and iron from mine-tailing leachate using chitosan hydrogels synthesized by gamma radiation. Environ. Earth Sci..

[60] Zhou G., Luo J., Liu C., Chu L., Crittenden J. (2018). Efficient heavy metal removal from industrial melting effluent using fixed-bed process based on porous hydrogel adsorbents. Water Res..

[61] Ma J., Liu Y., Ali O., Wei Y., Zhang S., Zhang Y., Cai T., Liu C., Luo S. (2018). Fast adsorption of heavy metal ions by waste cotton fabrics based double network hydrogel and influencing factors insight. J. Hazard. Mater..

[62] Jang J., Lee D.S. (2016). Enhanced adsorption of cesium on PVA-alginate encapsulated Prussian blue-graphene oxide hydrogel beads in a fixed-bed column system. Bioresour. Technol..

[63] Sharma N., Tiwari A. (2015). Nanomagnetite-loaded poly (acrylamide-co-itaconic acid) hydrogel as adsorbent for effective removal of Mn2+from contaminated water. Desalin. Water Treat..

[64] Lin P.J., Yang M.C., Li Y.L., Chen J.H. (2015). Prevention of surfactant wetting with agarose hydrogel layer for direct contact membrane distillation used in dyeing wastewater treatment. J. Membr. Sci..

[65] Wan J., Tao T., Zhang Y., Liang X., Zhou A., Zhu C. (2016). Phosphate adsorption on novel hydrogel beads with interpenetrating network (IPN) structure in aqueous solutions: Kinetics, isotherms and regeneration. RSC Adv..

[66] Yi Q., Tan J., Liu W., Lu H., Xing M., Zhang J. (2020). Peroxymonosulfate activation by three-dimensional cobalt hydroxide/graphene oxide hydrogel for wastewater treatment through an automated process. Chem. Eng. J..

[67] He S., Zhang F., Cheng S., Wang W. (2016). Synthesis of Sodium Acrylate and Acrylamide Copolymer/GO Hydrogels and Their Effective Adsorption for Pb^2+^ and Cd^2+^. ACS Sustain. Chem. Eng..

[68] Song X.H., Goh K.L., Wang K. (2019). The equilibrium and fixed-bed study of malachite green adsorption on chitosan hydrogels. Water Sci. Technol..

[69] Yan Y.Z., Zheng W., Huang D.Z., Xiao Z.Y., Park S.S., Ha C.S., Zhai S.R. (2020). Hierarchical multi-porous carbonaceous beads prepared with nano-CaCO_3_ in-situ encapsulated hydrogels for efficient batch and column removal of antibiotics from water. Microporous Mesoporous Mater..

[70] Pei Y.Y., Guo D.M., An Q.D., Xiao Z.Y., Zhai S.R., Zhai B. (2018). Hydrogels with diffusion-facilitated porous network for improved adsorption performance. Korean J. Chem. Eng..

[71] Bethi B., Manasa V., Srinija K., Sonawane S.H. (2018). Intensification of Rhodamine-B dye removal using hydrodynamic cavitation coupled with hydrogel adsorption. Chem. Eng. Process..

[72] Sharma N., Tiwari A. (2015). Nano-ZnO-loaded poly (acrylamide-co-itaconic acid) hydrogel as adsorbent for effective removal of iron from contaminated water. Desalin. Water Treat..

[73] Zhuang Y., Yu F., Chen J., Ma J. (2016). Batch and column adsorption of methylene blue by graphene/alginate nanocomposite: Comparison of single-network and double-network hydrogels. J. Environ. Chem. Eng..

[74] Gonçalves H., Fregolente P., de Andrade G., Maciel M.R., Fregolente L. (2021). Development of a Hydrogel Column for Water Removal from Fuels. Chem. Eng. Trans..

[75] Dong S., Wang Y., Li J., Zhang D., Zhou Y., Tong Y. (2020). Tuning the crosslink structure of cationic hydrogel for enhanced chromium(VI) removal: The covalent and electrostatic co-crosslinked effects and adsorption mechanism. Chem. Eng. J..

[76] He J., Cui A., Ni F., Deng S., Shen F., Yang G. (2018). A novel 3D yttrium based-graphene oxide-sodium alginate hydrogel for remarkable adsorption of fluoride from water. J. Colloid Interface Sci..

[77] Yang J., Chen X., Zhang J., Wang Y., Wen H., Xie J. (2021). Role of chitosan-based hydrogels in pollutants adsorption and freshwater harvesting: A critical review. Int. J. Biol. Macromol..

[78] Mohammadzadeh P.P., Peighambardoust S.J. (2018). A review on acrylic based hydrogels and their applications in wastewater treatment. J. Environ. Manag..

[79] Junlapong K., Maijan P., Chaibundit C., Chantarak S. (2020). Effective adsorption of methylene blue by biodegradable superabsorbent cassava starch-based hydrogel. Int. J. Biol. Macromol..

[80] Shalla A.H., Yaseen Z., Bhat M.A., Rangreez T.A., Maswal M. (2018). Recent review for removal of metal ions by hydrogels. Sep. Sci. Technol..

[81] Birgersson E., Li H., Wu S. (2008). Transient analysis of temperature-sensitive neutral hydrogels. J. Mech. Phys. Solids.

